# Shaping the Structure and Properties of Stellite 6 Alloy by Addition of Ti and W via Laser Cladding

**DOI:** 10.3390/ma18173968

**Published:** 2025-08-25

**Authors:** Jacek Górka, Tomasz Poloczek, Damian Janicki, Aleksandra Lont

**Affiliations:** Welding Department, Faculty of Mechanical Engineering, Silesian University of Technology, Konarskiego Street 18A, 44-100 Gliwice, Poland; damian.janicki@polsl.pl (D.J.); aleksandra.lont@polsl.pl (A.L.)

**Keywords:** Stellite 6, in situ, laser cladding, erosive wear, TiC, cobalt alloys

## Abstract

Cobalt-based alloys such as Stellite 6 are widely applied in demanding conditions because of their good resistance to wear, erosion, and corrosion, but further improvements in erosion resistance are still required. This work analyzes the effect of adding titanium and tungsten on the structure and properties of Stellite 6 coatings produced by laser cladding, aiming to enhance their erosion resistance. Penetrant tests confirmed that the additions did not reduce coating quality, and macroscopic observations showed that appropriate process parameters allowed for defect-free coatings with strong bonding to the substrate. Microstructural studies carried out by SEM/EDS (Scanning Electron Microscopy/ Energy Dispersive Spectroscopy) and XRD (X-ray Diffraction) revealed that the reference Stellite 6 coating consisted of a cobalt-based austenitic matrix with interdendritic chromium carbides, while Ti and W additions led to the in situ formation of primary and eutectic (Ti,W)C carbides. Transmission electron microscopy showed a gradient in tungsten concentration inside the primary carbides, with progressive tungsten dissolution into the TiC lattice. Although different powder compositions had only a moderate effect on hardness, erosion tests demonstrated that the coatings with Ti and W exhibited clearly improved resistance. In particular, the in situ carbides enhanced erosion resistance at 30° impingement angles, while also maintaining high resistance under 90° impact. These findings confirm that modifying Stellite 6 with Ti and W during laser cladding is an effective way to improve its durability in erosive conditions.

## 1. Introduction

The constant increase in demand for cobalt alloys in various industries is due to their remarkable resistance to erosive and abrasive wear as well as corrosion in different environments and their capability to operate at elevated temperatures, reaching 1000 °C. A widely used variety of cobalt alloy is the Co-Cr-W-C alloy which can be found under several names like Stellite 6, MetcoClad 6, Deloro 6, Weartech WT-6. It is mainly utilized as a coating material which can be deposited using plasma, gas flame, electric arc, or even laser beam [1,2,3,4]. Nowadays, advanced deposition methods such as a laser beam have gained special attention due to their distinct advantages such as highly controllable heat input and capability to limit thermal deformations with minimal crystallization times so that metastable phases can be formed [5,6,7]. Despite high prices of devices employing this technology, the numerous advantages they offer have generated interest in related technological processes. Due to the low heat input, a narrow heat-affected zone is formed with minimal dilution and with a strong bond between the coating and substrate, as demonstrated in studies provided by Cooper et al. [8]. Moreover, the accuracy in controlling the powder feed rate and cladding speed facilitates the obtaining of coatings with specific shapes and thicknesses, which helps minimize the need for additional machining required after processing [1,2]. Additionally, the short solidification time, resulting from the high temperature gradient between the substrate and the deposition area, promotes the formation of fine-grained microstructures, which exhibit superior service properties [9]. Finally, since the laser beam consists of photons and does not involve direct physical contact with the base material, it does not affect the chemical composition of the treated material. An emerging laser processing technology is the femtosecond–CW combined-pulse laser (CPL) technique, which significantly enhances absorption in the target material and enables efficient sputtering of molten material through shock-wave induction. Together with the spatial filamentation and transmission characteristics of the femtosecond laser, CPL presents a promising next-generation method for target damage, particularly in transparent, hard, and brittle materials [10].

Numerous studies have focused on analyzing the influence of laser processing parameters on the properties and microstructure of Stellite 6 coatings. Frenk et al. [11] successfully applied the Stellite 6 alloy onto austenitic stainless steel (X2CrNiMo 18-14), observing that higher scanning speeds resulted in finer secondary dendrite arm spacing. Obtained clads also exhibited better sliding wear resistance compared to the cast Stellite 6 alloy. Traxel et al. [12] successfully applied a Co-Cr-W-C alloy on cutting tool steel, obtaining clads characterized by the absence of defects such as porosity or cracks.

The beneficial properties of the Stellite 6 alloy come from its microstructure, which contains a γ-Co plastic matrix combined with eutectic M_7_C_3_ and M_23_C_6_ carbides. However, due to industry demands for reduced material costs and increased durability, there is a growing interest in further enhancing its erosion wear resistance [13,14,15,16]. One strategy to improve wear performance of Stellite 6 alloy is the in situ formation of carbides during the cladding process. This can be achieved by adding base powder elements like tungsten (W) or titanium (Ti), which have a strong affinity for carbide formation. Due to the formation of carbides during crystallization process, numerous advantages can be offered. With the in situ technique, there is no oxidation on the interfacial surfaces between the reinforcing particles and the matrix, which enhances ductility and mechanical properties of the coatings. Another positive aspect is that the reinforcing phases have high thermodynamic stability and do not dissolve at elevated temperatures. This indicates improved corrosion resistance along with a stronger interfacial bond between the matrix and the reinforcement. Using the in situ method also results with uniform distribution of the reinforcing phase which enhances mechanical properties [17,18,19,20,21].

Shahroozi et al. [22] investigated Stellite 6 alloy coatings reinforced with 10–40 wt% TiC using tungsten inert arc welding (TIG), finding that higher TiC concentrations led to enhanced hardness and wear resistance. Similarly, Acevedo et al. [23] demonstrated that the addition 2% of nano-TiC into Stellite 6 coatings significantly improved wear resistance. Wang et al. [13] reported a fivefold increase in wear resistance in Stellite 6 alloy reinforced with the addition of TiFe and Cr_3_C_2_, attributing the improvement to the formation of a TiC phase, which also raised the average hardness up to 658 HV. In another study, Wu et al. [24] explored the effects of Ti and Ni additions to Stellite 6, observing a transformation in carbide morphology from M_23_C_6_ eutectic carbides to separated TiC particles. The addition of titanium led to the segregation of chromium into the cobalt matrix by reacting with carbon, which reduced stacking-fault energy (SFE) and promoted ε-Co phase formation. Meanwhile, nickel additions increased the SFE, stabilizing the γ-Co phase. Study provided by Bartkowski et al. showed that addition of 30–60% WC to the Stellite 6 powder causes an increase in the microhardness but also a lower electrochemical corrosion resistance, where the overall wear resistance of this alloy is influenced by the volume fraction, size, shape, and distribution of carbides [25].

Although the correlation between the chemical composition of Stellite alloys and their erosion resistance has not been systematically established, several studies have provided valuable insights. Study provided by Nsoesie et al. [26] revealed that the erosion resistance of Stellite alloys comes from their high carbide content. In alloys with reduced carbon levels, intermetallic phases such as Co_3_Mo and CoMo_6_ form, which offer a similar strengthening effect as carbides, maintaining low erosion loss. They additionally identified a strong dependence between erosion resistance and the carbon content in the alloy. This relationship can be attributed to the increased resistance to microcutting, resulting from the rise in surface hardness with higher carbon content.

Samples containing fine Cr_7_C_3_ carbides exhibit better erosion resistance than those with relatively large (W,Co)C precipitates. Due to the brittleness of the large (W,Co)C reinforcing phases, they are more susceptible to cracking and chipping off from the matrix under continuous erodent impact.

In a separate investigation, Levin et al. [27] explored erosion behavior in cobalt-based superalloy clads, specifically Stellite 6 and Ultimet. They observed that under stable erosive wear conditions at elevated temperatures (400 °C), Stellite 6 had inferior erosion resistance compared to 316L stainless steel, Inconel-625, and the Ultimet alloy. Notably, they found no significant correlation between erosion loss and impingement angle. Instead, they concluded that at 400 °C, the erosion of Stellite 6 occurred primarily due to plastic deformation of the material. Research reported by Chen et al. [28] showed that Stellite 6 has erosion resistance that is over ten times better than the 1Cr11Ni2W2MoV steel with a hardness up to 455 HV which is caused by formation of hard carbide phases. They concluded that the Co matrix phase corrodes at a faster rate than the hard carbide phases, indicating that the carbides offer greater corrosion resistance.

Although prior studies have explored the influence of alloying elements or particle reinforcements on Stellite 6, there is still a lack of systematic understanding of how simultaneous Ti and W incorporation during laser cladding impacts in situ carbide formation, microstructural evolution, and erosion behavior. Addressing this gap is essential to optimize the design of advanced cobalt-based coatings with improved durability and cost efficiency for demanding industrial applications. The presented study focuses on the structure and property modification of cobalt-based alloys (Stellite 6) produced via laser cladding, with the main aim of enhancing their resistance to erosive wear. A comprehensive analysis was carried out to assess the influence of tungsten and titanium addition to the Co-Cr-W-C-Ti alloy on the possibility of in situ carbide formation. Particular emphasis was placed on the role of alloying elements in controlling the morphology and volume fraction of the reinforcing phases.

## 2. Materials and Methods

### 2.1. Materials and Laser Processing

For laser cladding processing, the following powders were used: Stellite 6 (spherical gas atomized powder Metcoclad 6, Oerlikon, Westbury, NY, USA), 99.8% W (spherical gas atomized powder GF89800599, Goodfellow, Cambridge, UK), 99.0% spherical Ti (Amperit 154, H.C. Starck, Goslar, Germany), and 99.5% C (1.04206.9025, Merck, Darmstadt, Germany). Prior to the cladding process, the powder mixtures were blended using a vertical planetary ball mill for 30 min, followed by drying at a temperature of 50 °C for 1 h to ensure uniformity and moisture removal. The detailed chemical composition of the powder mixtures are presented in Table 1. The chemical composition of the powders was selected based on preliminary tests, with the aim of optimizing the formation of the maximum volume fraction of carbide phases while avoiding any deterioration in coating quality (such as porosity, cracking, or the presence of unmelted powder particles). As a base material for laser processing, 10 mm thick S355JR structural steel (Cognor, Stalowa Wola, Poland) was used, which complies with the EN 10025-2 specification [29]. Before cladding, the substrate material was subjected to mechanical grinding, resulting in an average surface roughness of Ra = 1.6 μm and degreased using ethylic alcohol (Stanlab, Lublin, Poland).

**Figure 1:**
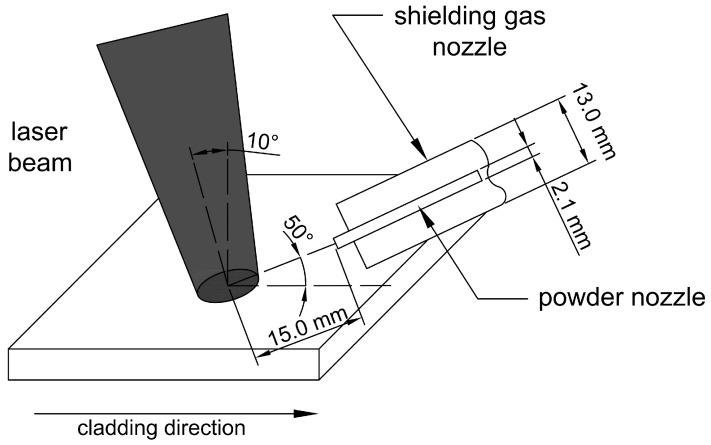
Schematic illustration of the laser cladding process [9].

The laser cladding process was provided on a solid-state laser TruDisk 3302 (TRUMPF, Ditzingen, Germany) equipped with a computer-controlled positioning system. For the laser processing, a disk-type powder feeding system with an accuracy of up to 0.1 g/min was used. Based on the geometrical analysis of preliminary samples, the laser beam was defocused by increasing the focal length by 30 mm to minimize excessive substrate penetration and to enlarge the heated surface area. The laser head was inclined at an angle of 10° from the vertical axis, while the powder was delivered into the molten pool through a cylindrical nozzle with a diameter of 2.1 mm, positioned 15 mm from the deposition zone. A schematic illustration of the laser cladding process is provided in Figure 1. High-purity argon gas (99.999%) served as the shielding and powder carrier gas, with respective flow rates of 15 L/min and 3 L/min. Before laser processing, the samples were not subjected to preheating, and during cladding, the inter-pass temperature was controlled and maintained below 50 °C. Each sample consisted of four single passes with a 50% overlap. Through a series of preliminary experiments, the process parameters were optimized and determined to be a laser power of 1750 W, a powder feed rate of 35 mg/min, and a scanning speed of 200 mm/min, providing the best compromise between deposition quality and microstructural homogeneity.

### 2.2. Macro- and Microstructural Examination with Penetrant Testing

Microscopic examination was conducted using a Phenom World Pro scanning electron microscope (SEM, Thermo Fisher Scientific, Waltham, MA, USA) coupled with energy-dispersive X-ray spectroscopy (EDS). The surface fraction of individual phases was quantified by applying the planimetric method across six distinct regions per sample, including areas subjected to secondary thermal cycles.

Transmission electron microscopy (TEM) analysis was carried out using a Titan 80/300 microscope (FEI, Hillsboro, OR, USA), combined with high-resolution TEM (HRTEM) and scanning TEM (STEM) techniques. Phase identification was performed using selected-area electron diffraction (SAED) patterns. Thin foils for TEM examination were prepared using xenon plasma focused ion beam (Xe-PFIB) technology (Helios G4, Thermo Fisher Scientific, Waltham, MA, USA). Additionally, phase composition was analyzed via X-ray diffraction (XRD) using a PANalytical X’Pert PRO MPD diffractometer (Malvern Panalytical, Malvern, UK) equipped with a cobalt anode. Diffraction data were acquired in continuous scan mode over a 2θ range of 25° to 130°, with a step size of 0.1444° and a counting time of 0.026 s per step.

Macroscopic evaluations were conducted using a stereoscopic optical microscope (SZX9, Olympus, Tokyo, Japan). Sample geometrical parameters from macrographs were determined using AutoCAD 2024 software (Autodesk, San Francisco, CA, USA), where the coating dilution was calculated according to Equation (1) where *F_BM_* represents the cross-sectional area of the melted substrate and *F_c_* denotes the cross-sectional area of coating.

(1) $$U=\frac{F_{BM}}{F_{BM}+F_{C}}\times 100\;[\%]$$

Penetrant tests were conducted using the color contrast technique, with a dwell time of 10 min and development time of 15 min. The procedure utilized MR 79 cleaner, 68 NF penetrant, and MR 70 developer (MR Chemie, Unna, Germany). Prior to testing, samples were rinsed using MR79 cleaner to eliminate surface contaminants. The testing temperature, measured with a pyrometer, was 22 °C, while the light intensity during examination was 920 lux. Penetrant observations were performed at a distance of 200 mm from the sample with an observation angle of 0°.

**Figure 2:**
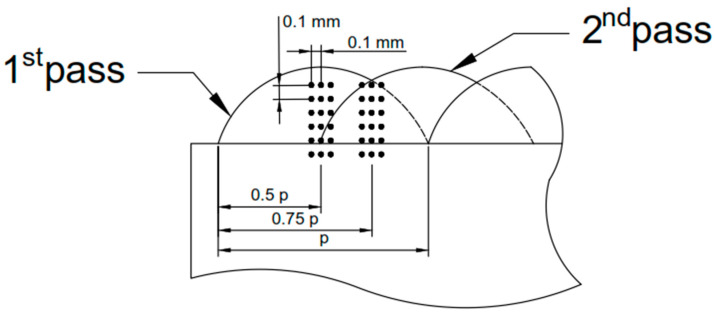
Microhardness schematic diagram.

Vickers microhardness measurements were performed on the cross-sections of the coatings using a Wilson Wolpert 401 MVD hardness tester (Wilson Instruments, Instron Company, Norwood, MA, USA). The tests were conducted under a 300 g load with a dwell time of 10 s. Measurements were taken along six lines: three aligned with the axis of the laser pass and three located in the inter-pass regions (which were under subjected and secondary thermal cycles). Each set of measurements was performed with a spacing of 0.1 mm between adjacent lines, as shown in Figure 2.

**Figure 3:**
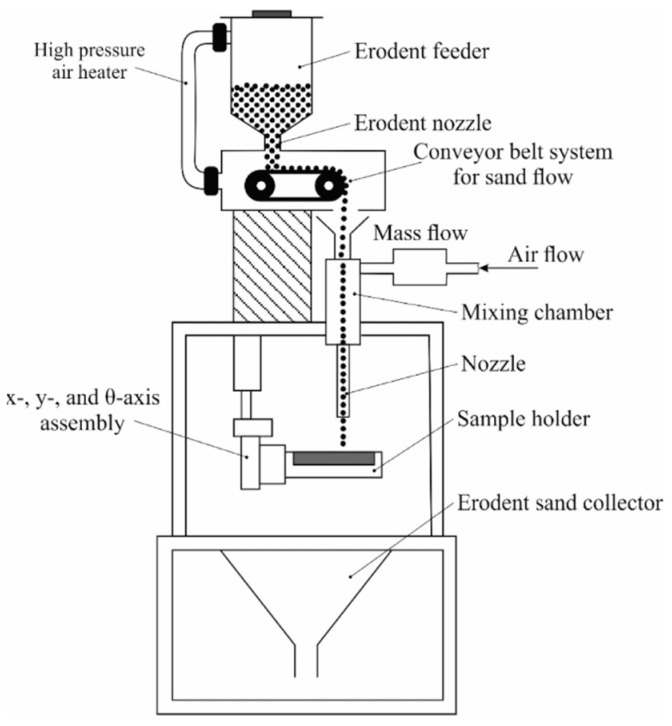
Schematic diagram of erosion resistance test rig [31].

Erosion resistance tests were performed on a test rig compliant with ASTM G76-04 standard. A schematic diagram of the testing system is presented in Figure 3. Before the tests, the surfaces of the samples were ground to achieve a surface roughness of Ra = 0.5 µm. During the test, particles of Al_2_O_3_ with a 50 µm grain size were carried in a dry air stream at a velocity of 70 m/s, with a 2 g/min powder feed rate. An erodent nozzle was positioned 10 mm above the samples’ surfaces. The tests were performed at two impingement angles: 30° and 90°, with 10 min duration of each test. Mass loss measurements were obtained using a WAX 60/220 analytical balance (Radwag, Radom, Poland), which provides a measurement accuracy of 0.0001 g. The erosion rate and erosion value parameters were calculated in accordance with the ASTM G76-04 standard [30], using Equations (2) and (3), respectively.

(2) $$Erosion\;rate=\frac{mass\;loss\;[g]}{test\;time\;[min]}$$

(3) $$Erosion\;value=\frac{volume\;loss\;[{mm}^{3}]}{total\;mass\;of\;abrasive\;[g]}$$

## 3. Results and Discussion

### 3.1. Penetrant Tests

**Figure 4:**
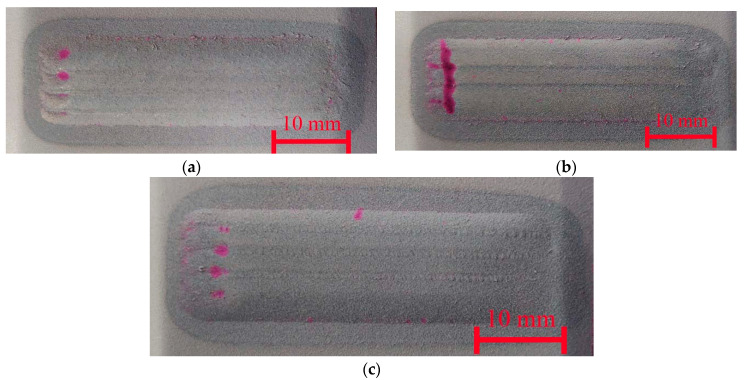
Samples after penetrant test: (**a**) M6; (**b**) M6Ti; (**c**) M6TiCW; designations according to Table 1.

The penetrant tests, Figure 4, revealed false indications in all samples in the regions where the cladding process was carried out. This is attributed to the presence of craters containing unmelted powder particles in these areas, which hinder the removal of excess penetrant before application of the developer. An observed indication on the M6Ti sample, oriented perpendicular to the cladding direction, corresponds to a crack resulting from the high cooling rates at the end of the cladding process. Rapid cooling, caused by the abrupt cessation of laser radiation, led to increased thermal stresses, which ultimately caused the crack at the end of the coating [13,32]. The observed indication is localized in the termination area of the clad and does not affect the surface integrity in regions where the process proceeded under stable conditions. In the M6TiCW sample, a 1.5 mm linear indication was detected at the midpoint of the first pass. The powder composition used for the coating exhibited a high carbon and tungsten content, promoting the formation of hard carbide phases. These phases contribute to high internal stresses which, when combined with the rapid heating and cooling cycle during the cladding process, can lead to the formation of cracks. The identified indication is minor in size and does not propagate into subsequent tracks, indicating the high quality of coating.

### 3.2. Macrostructure

**Figure 5:**
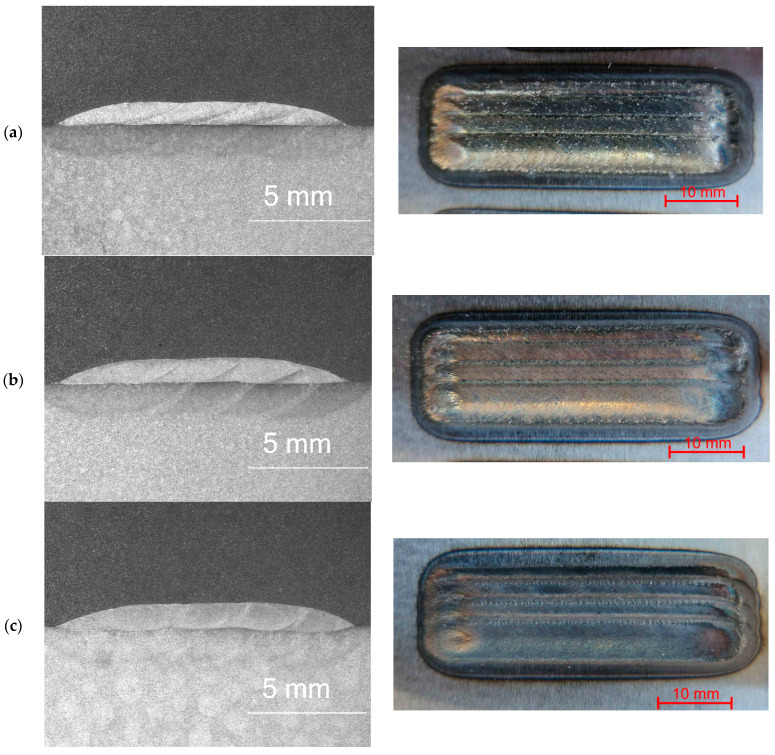
Macrostructure of the coatings on cross-section and their surface overview: (**a**) M6; (**b**) M6Ti; (**c**) M6TiCW. Designation in accordance with Table 1.

Figure 5 presents the macrostructures of the produced coatings on a cross-section, aiming to determine geometrical parameters of samples. All fabricated coatings exhibited high quality, indicating the stability of the laser processing. The coatings were characterized by a clean metallic surface, which indicates effective shielding gas protection that prevents the oxidation of the coatings. Slight overheating of the base material was observed in the zones surrounding the coatings.

The produced coatings, regardless of their chemical composition, were free from typical defects such as porosity and lack of fusion or cracks, which are commonly observed in high-hardness materials reinforced by the ex situ method. The applied overlap of 50% allowed for the formation of coatings with low surface waviness, which is an important factor in reducing post-process machining [33]. Macrostructural analysis of the multi-pass cladded layers revealed that the depth of the melted substrate material was consistently highest for the first track of each coating. This can be attributed to the cladding technique, where the substrate material is subjected to greater heat exposure during the first pass, while each subsequent track, due to the applied overlap, partially remelts the previously deposited material.

The cross-sectional area, width, and height of the coatings increased with the rising graphite content in the powder mixtures, as seen in Table 2. This increase in the cross-sectional area of the coating may be attributed to the enhanced absorption of laser radiation by carbon [34,35], leading to a larger molten pool and higher melted substrate area. The amount of powder melted and incorporated into the coating is directly dependent on the size of the molten pool. Therefore, an increase in molten pool area contributes to higher powder utilization efficiency. The in situ reaction between molten titanium and carbon which occurs during the cladding process is an exothermic reaction. Consequently, increasing the content of titanium and carbon results in greater heat generation within the molten metal pool. According to studies by Janicki [36], the calculated energy of formation for TiC (at 15 vol.%) during laser alloying was approximately 18 J/mm. Compared to the linear energy within the tested cladding parameters (525 J/mm), this value is relatively small and should not significantly affect the process stability. For the M6 sample, the dilution was 1.6%, which is very low for laser cladding processes. Furthermore, no evidence of lack-of-fusion defects at the substrate–coating interface was observed, as confirmed by microscopic analysis. As the content of alloying additions in the powder mixtures increased, a corresponding increase in substrate material dilution was noted, with the highest value recorded for the M6TiCW coating at 12.5%.

### 3.3. Microstructure

**Figure 6:**
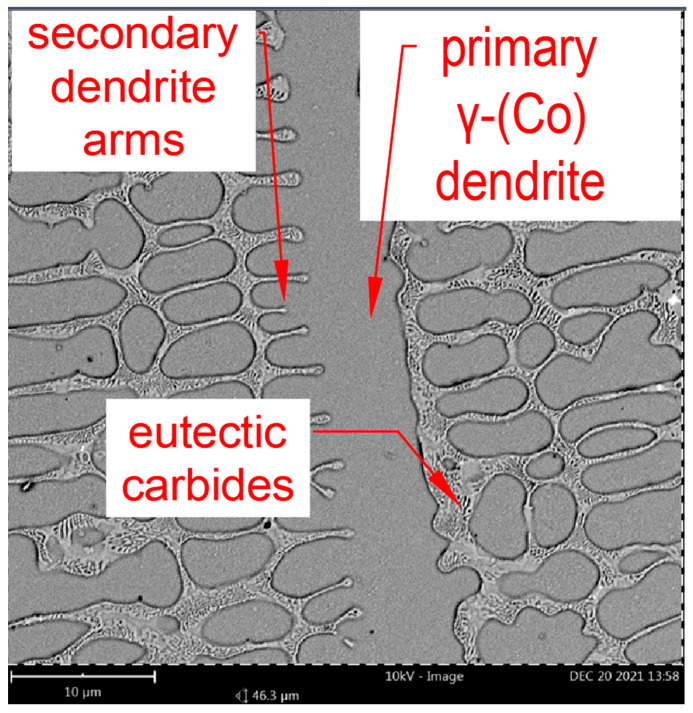
M6 coating microstructure.

**Figure 7:**
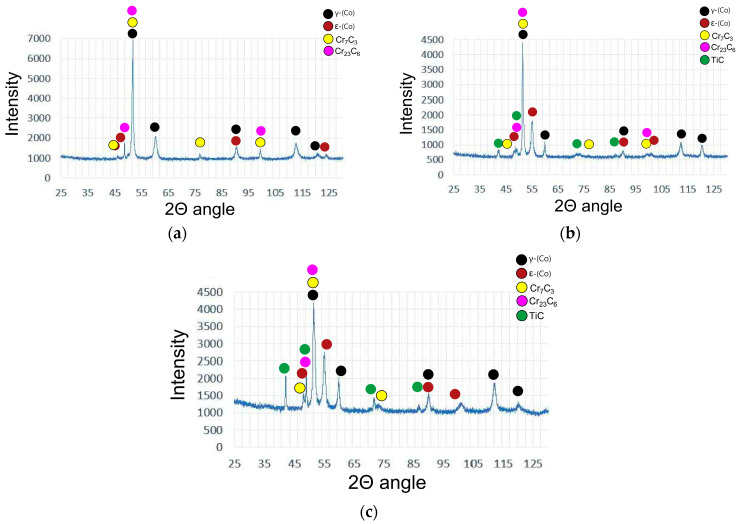
XRD results: (**a**) M6; (**b**) M6Ti; (**c**) M6TiCW. Labeled according to Table 1.

Figure 6 presents a microstructure image of the M6 coating deposited using a commercial Co-Cr-W-C alloy (Stellite 6 type), which enables a comparative analysis of structural changes influenced by the addition of alloying elements (Ti,W). Microstructural examination of the base M6 coating indicates that, in the initial stage, solidification occurs through the formation of γ-(Co) solid solution dendrites, which crystallize in a face-centered cubic (FCC) structure. These dendrites can be observed reaching lengths of up to 300 μm, with their growth direction aligned with the heat flow direction. Their nucleation initiates at the fusion line and progresses toward the surface of the coating. From the described columnar dendrites, the growth of perpendicular secondary dendrite arms can be observed. These secondary arms play a significant role in assessing the cooling rate during the laser cladding process. Based on the research conducted by Frenk et al. [11], a correlation between the cooling rate and secondary arm spacing was established. To calculate the average cooling rate, the secondary arm spacing was measured in six distinct regions of the M6 coating, with five measurements taken in each region. The average cooling rate value of the M6 coating was approximately 800 K/s. In the interdendritic regions, plate-like precipitates of Cr_7_C_3_ and Cr_23_C_6_ eutectic carbides were observed, as confirmed by the X-ray phase analysis in Figure 7. XRD analysis reveals that, upon continued cooling, the γ-(Co) phase partially transforms into the ε-(Co) phase, which crystallizes in a hexagonal close-packed (HCP) structure, as seen in Figure 7. These findings are consistent with previously reported data concerning the behavior of Co-Cr-W-C alloys under cooling [37,38].

**Figure 8:**
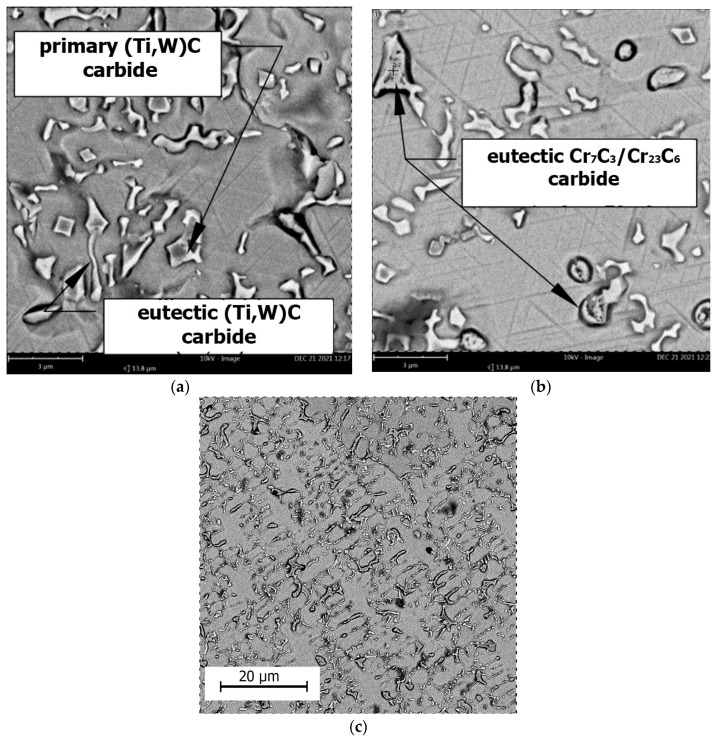
SEM M6Ti sample results: (**a**) Morphology of primary and eutectic (Ti,W)C carbides; (**b**) Precipitates of Cr_7_C_3_ and Cr_23_C_6_ eutectic carbides; (**c**) Microstructure of the coatings at a magnification of 4000×.

As anticipated, the addition of titanium resulted in a significant alteration of the microstructure of the M6Ti sample through the formation of primary carbides which are produced by an in situ reaction. The average volume fraction of these precipitates within the coating was 8.0 ± 1.0%, as presented in Table 3. Due to strong titanium affinity for carbon, the in situ synthesis of TiC carbides takes place directly within the molten pool, as shown in Figure 8a. The carbon and titanium concentration (1.1 wt.% C and 4.0 wt.% Ti) enabled the crystallization of titanium carbide as a primary phase. The Co–Ti–C ternary phase diagram, provided in research published by Bandyopadhyay et al. [39], suggests that the observed carbides may form via a eutectic reaction. However, considering the presence of multiple alloying elements and the non-equilibrium solidification conditions, the TiC precipitates are formed mainly as a primary phase. This is evidenced by the cubic morphology of the TiC precipitates and their occurrence within the primary γ-(Co) dendrites, rather than being confined to the eutectic regions. The presence of primary TiC precipitates served as nucleation sites for the growth of cobalt-based solid solution matrix dendrites, contributing to grain refinement of the microstructure (Figure 8c). Following the crystallization of primary carbides, dendrites of the γ-(Co) phase nucleate and grow within the microstructure. These dendrites subsequently undergo a partial transformation into the ε-(Co) phase, which was confirmed by X-ray diffraction analysis (Figure 7). Additionally, the primary carbides act as nucleation substrates for eutectic TiC carbides, which exhibit a morphology of laths approximately 1 μm in length. The average volume fraction of eutectic TiC precipitates in the coating was 5.3 ± 1.0%, as presented in Table 3. The addition of titanium to sample M6Ti (with carbon content maintained at 1.1 wt%) led to a reduction of eutectic chromium carbides to very low value (0.6 ± 0.3 vol.%). This effect is attributed to titanium’s higher affinity for carbon compared to chromium, which suppresses the formation of eutectic chromium carbides. As a result, the cobalt-based matrix retains a higher chromium content.

**Figure 9:**
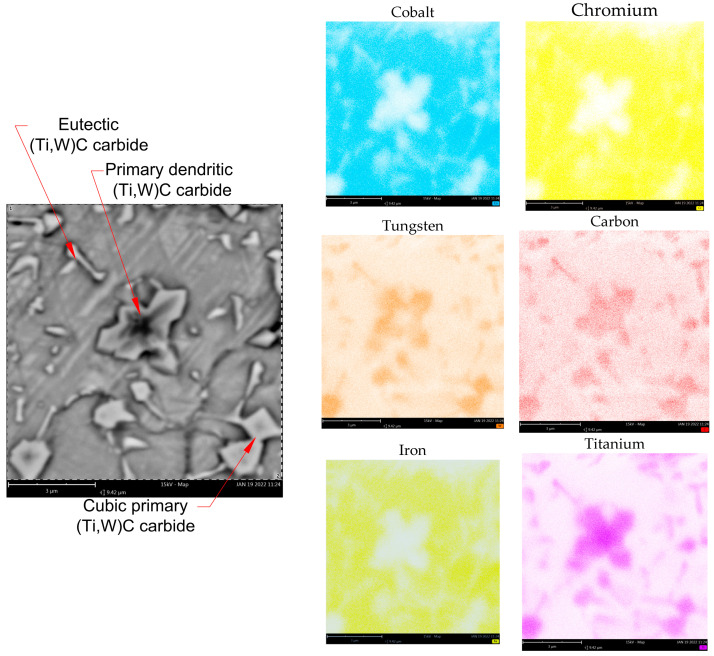
Microstructure of the M6TiCW coating in the central region of the cross-section with EDS elemental distribution maps.

As demonstrated on EDS maps (Figure 9), both primary and eutectic (Ti,W)C carbides dissolve tungsten into their composition, leading to a lower concentration of this element in the matrix. The study presented by Kapoor [40] indicates that tungsten is a key element responsible for solid solution strengthening of the matrix due to its large atomic size, which hinders dislocation motion. Consequently, the absence of tungsten in the matrix may ultimately reduce erosion resistance. The M6TiCW sample chemical composition was designed with an increased tungsten content of 5.9 wt.%. Additionally, elevated amounts of titanium (8.0 wt.%) and graphite (3.0 wt.%) were added to enhance the volume fraction of (Ti,W)C carbide phases in the alloy microstructure.

The microstructure of the M6TiCW alloy was characterized by a high-volume fraction (12.2 ± 0.3 vol.%) of primary (Ti,W)C carbides with a cubic morphology. Scanning electron microscopy analysis revealed a minor presence of primary (Ti,W)C carbides characterized by dendritic morphology, as shown in Figure 9. The largest primary (Ti,W)C carbides were observed near the surface region, reaching sizes up to 5 μm. In the central region and near the fusion line of coating, a reduction in the size of the primary carbides was observed, with their average size decreasing to 1–2 μm. High titanium and carbon contents promoted the formation of eutectic (Ti,W)C carbides with a lamellar morphology, with an average volume fraction of 6.1 ± 0.5%. The elevated concentrations of titanium and tungsten suppressed the formation of eutectic chromium carbides, as shown in M6Ti sample. Despite the high content of graphite, titanium, and tungsten, combined with rapid solidification rates, no unmelted powder particles were observed in the microstructure. This indicates complete in situ reaction of carbon, resulting in the successful in situ synthesis of carbide phases.

**Figure 10:**
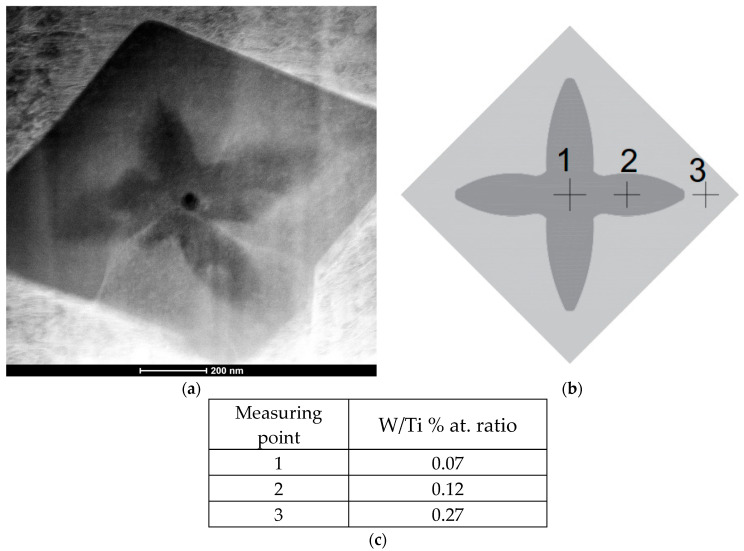
Chemical analysis of (Ti,W)C carbide: (**a**) STEM HAADF image of carbide; (**b**) Morphological sketch of the carbide; (**c**) W/Ti % at. ratio at various measurement points.

**Figure 11:**
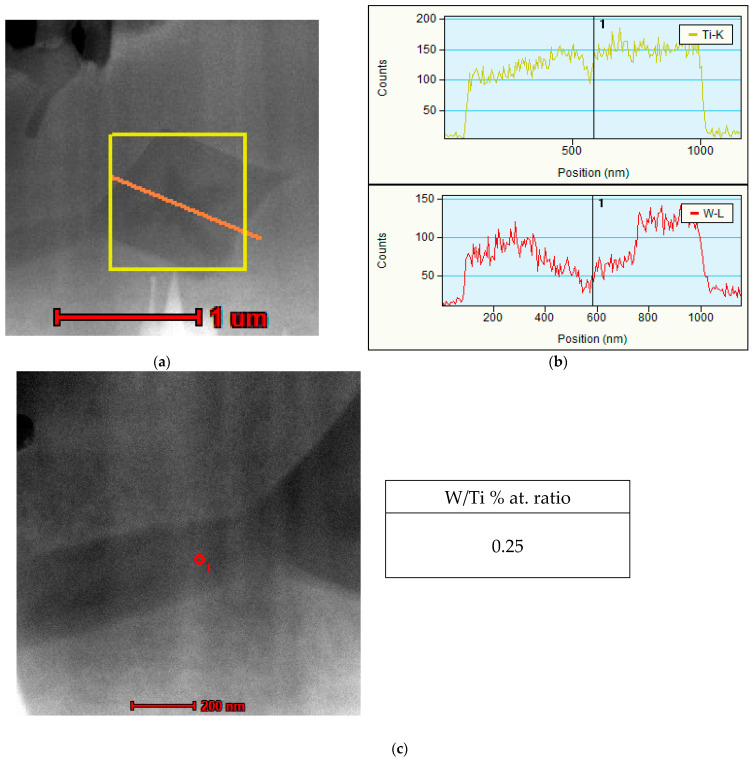
TEM image: (**a**) primary (Ti,W)C carbide with the measurement line; (**b**) line scan analysis of titanium and tungsten concentrations across the carbide; (**c**) eutectic (Ti,W)C carbide with titanium and tungsten point ratio analysis.

Additionally, an analysis of the primary (Ti,W)C carbides was conducted using transmission electron microscopy (TEM). The results revealed a gradient concentration of tungsten in the primary carbides. Specifically, a lower tungsten content was observed in the core (with a W/Ti atomic ratio of 0.07), while the outer layer exhibited a significantly higher tungsten concentration (W/Ti atomic ratio of 0.27), as shown in Figure 10. This behavior can be attributed to the higher affinity of titanium for carbon compared to tungsten [41] Line scans performed across the (Ti,W)C carbide confirmed this tungsten gradient concentration with a uniform distribution of titanium, as demonstrated in Figure 11. In summary, during cooling at the first stage of nucleation, primary carbides form at lower concentrations of tungsten. However, as growth progresses, more tungsten atoms dissolve into the titanium carbide lattice, leading to a compositional gradient of tungsten. Gibbs free energy values for TiC are lower than for WC carbides, indicating that the probability of TiC phase precipitation from the molten metal pool is higher [41,42], which is consistent with the observations.

Furthermore, chemical composition point analysis of the eutectic (Ti,W)C carbides (Figure 10) indicated that the concentrations of tungsten and titanium were similar to those found in the outer layer of the cubic primary (Ti,W)C carbide, as can be seen in Figure 11.

### 3.4. Microhardness

**Figure 12:**
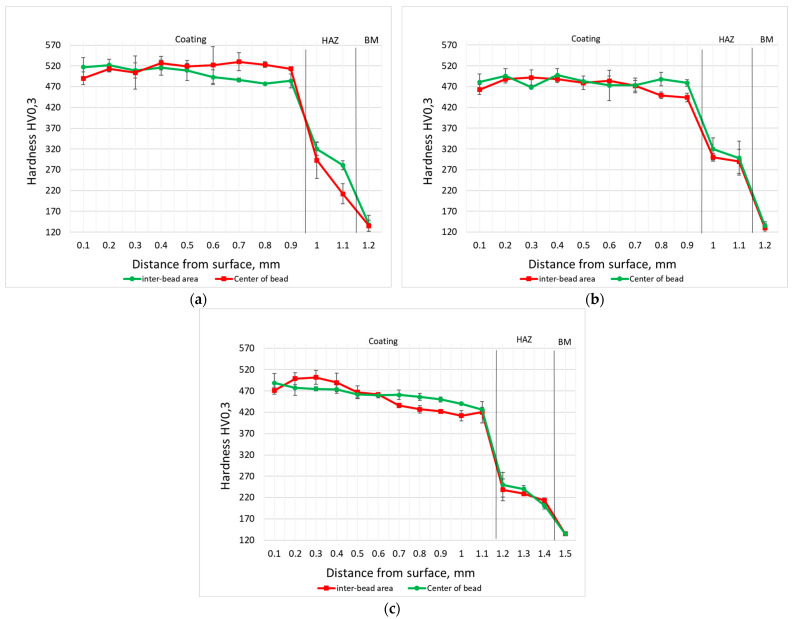
Microhardness: (**a**) sample M6; (**b**) sample M6Ti; (**c**) sample M6TiCW. Designation in accordance with Table 1 (HAZ—heat-affected zone; BM—base material).

The Vickers hardness values of the coatings on the cross-section are presented in Figure 12. The obtained data indicate only slight variations in hardness despite changes in the chemical composition of samples produced during laser processing. The M6 sample microstructure consists of cobalt-based solid solution dendrites and Cr_7_C_3_ and Cr_23_C_6_ eutectic carbides, with a uniform distribution of this phase within the alloy. This directly contributes to the high hardness of the sample (469 to 530 HV0.3). The described carbide phases exhibit high hardness, with Cr_23_C_6_ and Cr_7_C_3_ reaching 13.2 GPa and 18.2 GPa, respectively [43]. The M6Ti sample contains an addition of titanium, and the measured hardness values were comparable to those of the base M6 sample, ranging from 451 to 516 HV_0.3_. Despite the presence of (Ti,W)C carbides, which are characterized by high hardness and a uniform distribution within the alloy microstructure, no significant increase in the hardness parameter was observed. A further increase in the content of carbon (3.0 wt.%), titanium (8.0 wt.%), and tungsten (5.9 wt.%) in M6TiWC sample resulted in a slight decrease in hardness, with an average value of 458 HV0.3. Although the reinforcing phase in the coating microstructure consists of high volume fraction (18.1 vol.% of (Ti,W)C carbides), no eutectic phase consisting of the Cr_23_C_6_ or Cr_7_C_3_ phases were observed. The absence of these carbides contributes to the observed reduction in coating hardness. The hardness analysis of the inter-pass region in the M6 sample revealed a slight decrease in hardness near the fusion line, which is attributed to the locally coarse-grained structure resulting from the heat input generated by the subsequent cladding pass. For the remaining samples (M6Ti and M6TiCW), no significant differences in hardness were observed between the center of individual tracks and the inter-pass regions due to the high thermal stability of (Ti,W)C phases. The hardness in the heat-affected zone (HAZ) for all samples ranged from 180 to 332 HV_0.3_, subsequently stabilizing in the base material at values between 120 and 130 HV_0.3_.

### 3.5. Solid Particle Erosion Tests

Erosion tests demonstrated that by appropriately controlling the chemical composition of the Co-Cr-W-C-Ti alloy, it is possible to shape its resistance to erosive wear. The occurrence of carbide phases through in situ synthesis of (Ti,W)C significantly enhanced the erosion resistance compared to the unmodified base Stellite 6 alloy. The M6TiCW sample, containing 18.3 vol.% of (Ti,W)C phases, exhibited reduction in average erosion value at a 30° impingement angle, while maintaining a stable erosion value at a 90° angle compared to the reference M6 coating, as seen in Table 4.

For the M6 and M6Ti samples, a lower erosion rate was observed at a 90° impingement angle, indicating that the wear behavior is characteristic for ductile materials, where, under perpendicular impact of the erodent surface, a strengthening mechanism occurs due to the work hardening [44,45]. Sample M6TiCW with a high volume fraction of (Ti,W)C carbides exhibited similar erosion rate regardless of the impingement angle. A significant difference in average erosion value was observed for the M6Ti sample at a 90° impingement angle, where a 28% reduction (0.07 mm^3^/g) was recorded compared to the reference coating (M6), as shown in Table 4. The addition of titanium promoted the formation of fine, uniformly distributed (Ti,W)C primary carbides, which suppressed the growth of eutectic chromium carbides, contributing to a higher material loss during erodent impact. The suppression of eutectic chromium carbide formation in sample M6Ti led to an increased erosion rate at a 30° impingement angle, resulting from the intensified microcutting mechanisms.

**Figure 13:**
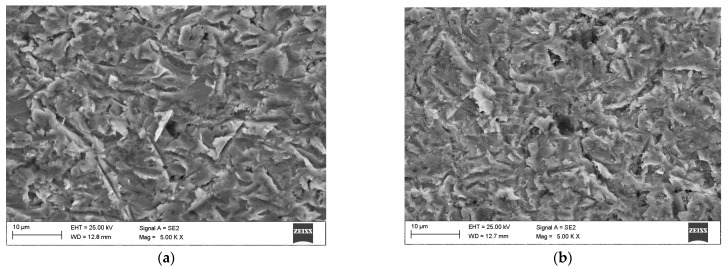

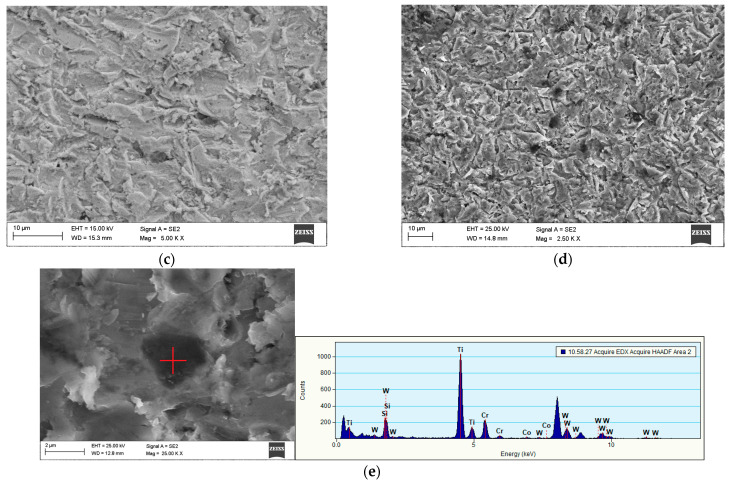
SEM surface after erosion tests: (**a**) sample M6Ti 30° impingement angle; (**b**) sample M6Ti 90° impingement angle; (**c**) sample M6TiCW 30° impingement angle; (**d**) sample M6TiCW 90° impingement angle; (**e**) sample M6TiCW with chipped (Ti,W)C carbide combined with an EDS diffractogram.

Analysis of the results obtained from erosion tests at a 30° impingement angle reveals significant changes in the average erosion value. Compared to the reference coating (M6), a decrease of 35% in the average erosion value was observed (0.013 mm^3^/g) for the M6TiCW coating. A higher volume fraction of titanium carbides, along with the presence of eutectic chromium carbides in the M6TiCW sample, did not markedly influence the erosion rate at a 90° impingement angle but did reduce the severity of microcutting (Figure 13a) occurring at a 30° angle. In this case, the kinetic energy of the erodent particles was dissipated upon impact with hard carbide phases or through rebound. In contrast, the M6Ti sample exhibited the highest average erosion value, showing an increase of 30% relative to the reference sample.

The studied metal-matrix composite coatings, characterized by a complex microstructure comprising a metallic matrix reinforced with (Ti,W)C, exhibit two distinct mechanisms of erosive wear. The first is related to plastic deformation of the material matrix, which is evidenced by grooves and a plastically deformed fragment (Figure 13), which is the typical mechanism of wear for ductile materials [46]. The second is caused by cracking and chipping of the hard carbide phases (Figure 13e), which is characteristic for brittle materials. The present study did not identify a definitive correlation between the hardness of the coatings and their erosion resistance.

## 4. Conclusions

Based on the results of the present study on shaping the structure and properties of cobalt-based alloys (Co-Cr-W-C type) through the addition of titanium and variation in carbon and tungsten content, the following conclusions can be drawn:

- Laser cladding of Co-Cr-W-C-Ti alloys produced defect-free coatings, where increased graphite and titanium content enhanced laser absorption, leading to greater coating thickness and higher dilution.
- The addition of titanium and increased carbon and tungsten content to the base Co-Cr-W-C alloy enabled the in situ formation of primary and eutectic (Ti,W)C carbides, where despite the formation of hard (Ti,W)C carbides, different powder compositions for laser cladding had only a moderate influence on the coating hardness.
- Transmission electron microscopy revealed a tungsten concentration gradient within primary (Ti,W)C carbides, indicating that TiC primary precipitates initially form at low tungsten levels (0.07 W/Ti % at. ratio) with progressively more tungsten dissolving into the carbide lattice reaching a W/Ti atomic ratio of 0.27 in the outer layer.
- Results from erosion testing showed that the inclusion of C and Ti to the Co-Cr-W-C alloy significantly influences erosive wear. The in situ synthesis of (Ti,W)C carbides enhanced the erosion resistance compared to the base alloy (M6 sample) at an impingement angle of 30°, while maintaining high erosion resistance at a 90° angle.

## Figures and Tables

**Table 1:** Chemical compositions of powder mixtures.

Sample Designation	C	Co	Cr	Fe	Mn	Mo	Ni	Si	W	Ti
M6	1.1	balance	27.3	0.1	0.1	0.1	0.8	1.6	4.4	-
M6Ti	1.1	balance	26.2	0.1	0.1	0.1	0.8	1.5	4.2	4.0
M6TiCW	3.0	balance	24.0	0.1	0.1	0.1	0.7	1.4	5.9	8.0

**Table 2:** Geometrical parameters of coatings.

Sample Designation	Width (mm)	Height (mm)	Cross-Sectional Area of the Melted Substrate (mm^2^)	Cross-Sectional Area of the Coating Buildup (mm^2^)	Cross-Sectional Area (mm^2^)	Dilution (%)
M6	12.0 ± 0.1	0.94 ± 0.05	0.15 ± 0.05	9.12 ± 0.12	9.27 ± 0.17	1.6
M6Ti	12.1 ± 0.1	0.96 ± 0.03	0.41 ± 0.10	9.52 ± 0.15	9.93 ± 0.25	4.2
M6TiCW	12.6 ± 0.2	1.01 ± 0.03	1.39 ± 0.12	9.73 ± 0.15	11.12 ± 0.27	12.5

**Table 3:** Average volume fraction.

Sample Designation (Acc. to Table 1)	Average Volume Fraction of Primary (Ti,W)C Carbides [%]	Average Volume Fraction of Eutectic (Ti,W)C Carbides [%]	Average Volume Fraction of Co-Based Solid Solution [%]	Average Volume Fraction of Eutectic Consisting of M_7_C_3_/M_23_C_6_ Carbides [%]
M6	-	-	65.3 ± 0.7	34.7 ± 0.7
M6Ti	8.0 ± 1.0	5.3 ± 1.0	86.1 ± 1.7	0.6 ± 0.3
M6TiCW	12.2 ± 0.3	6.1 ± 0.5	81.7 ± 0.7	-

**Table 4:** Solid particle erosion test results.

Sample Designation (Acc. to Table 1)	Average Erosion Rate [mg/min]		Average Erosion Value [mm^3^/g]	
Impingement Angle	30°	90°	30°	90°
M6	0.020 ± 0.001	0.015 ± 0.001	0.34 ± 0.02	0.25 ± 0.02
M6Ti	0.026 ± 0.002	0.011 ± 0.002	0.42 ± 0.03	0.18 ± 0.02
M6TiCW	0.013 ± 0.002	0.016 ± 0.001	0.21 ± 0.02	0.24 ± 0.02

## Data Availability

The original contributions presented in the study are included in the article, and further inquiries can be directed to the corresponding author.
